# Three-month Delay in Rotator Cuff Repair: 2-year Follow-up

**DOI:** 10.5435/JAAOSGlobal-D-23-00283

**Published:** 2024-01-22

**Authors:** Christopher Clinker, Karch M. Smith, Hiroaki Ishikawa, Christopher Joyce, Robert Z. Tashjian, Peter N. Chalmers

**Affiliations:** From the Department of Orthopaedic Surgery, University of Utah, Salt Lake City, UT (Mr. Clinker, Dr. Ishikawa, Dr. Joyce, Dr. Tashjian, Dr. Chalmers); Department of Orthopaedic Surgery, University of California San Diego, San Diego, CA (Dr. Smith).

## Abstract

**Introduction:**

This study examined 2-year outcomes of patients who underwent delayed rotator cuff repair (RCR) compared with those who underwent RCR without delay.

**Methods:**

In this prospective comparative study, two groups were formed: (1) patients planning RCR during a 6-week elective surgery ban and (2) patients undergoing RCR at least 6 weeks after the ban. The Simple Shoulder Test, American Shoulder and Elbow Surgeon score, and visual analog scale for pain were collected preoperatively and at 2 years postoperatively. Magnetic resonance imaging assessed healing 6 months postoperatively.

**Results:**

With a 93.3% 2-year follow-up (13/15 delay group, 15/15 control), there was an 87-day difference in presentation to surgery (*P* = 0.001), with no significant preoperative demographic or tear characteristic differences between groups. Intraoperatively, there were no differences between groups in repair characteristics. Preoperative versus postoperative differences in American Shoulder and Elbow Surgeon score (*P* < 0.001), visual analog scale (*P* < 0.001), and Simple Shoulder Test scores (*P* < 0.001) were significant but not between groups (*P* = 0.650, 0.586, 0.525). On MRI, 58% in the delay group and 85% in the control group had healed (*P* = 0.202).

**Discussion:**

Although a 3-month delay showed no statistically significant effect on outcomes, the delay group had an approximately 27% higher failure rate. Thus, although a 3-month period of nonsurgical treatment before RCR may be reasonable, larger studies are warranted for definitive conclusions.

Rotator cuff tears (RCTs) occur in up to 30 to 40% of those aged older than 65 years.^1^ Tears can be managed either nonsurgically or with surgical rotator cuff repair (RCR). Often, nonsurgical treatment, such as physical therapy, will be attempted before RCR, in the hopes of avoiding the lengthy recovery period and potential risks.^2,3^ However, many patients fail nonsurgical treatment, and RCR has been found to have superior outcomes in both randomized clinical trials and comparative studies.^4,5^

It remains unclear whether delaying RCR to allow a trial of nonsurgical treatment has any adverse consequences if subsequent RCR is performed. Animal studies have had conflicting conclusions with some studies demonstrating even a 12-week delay in surgical treatment could lead to loss of muscle, tuberosity bone, and deterioration in the biomechanical properties of the tendon.^6,7^ However, Koike et al.^8^ demonstrated in another animal study that a 12-week delay did not impair enthesis formation. Clinical studies have had similar confounding results as some retrospective studies have demonstrated that neither a 3-month delay nor a 6-month delay in surgical treatment negatively influenced outcomes, and it may take up to 18 months before muscular atrophy occurs.^5,9-11^ However, other clinical studies have shown that a delay in surgical treatment is associated with significantly worse outcomes than those who do not have a delay in surgery.^5,12,13^

Addressing this question without notable bias is challenging. Retrospectively, delays in surgical treatment can be related to a variety of reasons such as medical comorbidities or social issues, which can secondarily influence the surgical outcomes.^14^ Prospectively, few patients will consent to be randomized to a delay in treatment, so any randomized study would suffer notable selection bias. However, in March 2020, the COVID-19 pandemic led to a 6-week suspension of elective surgeries at our facility. During this period, no RCRs were performed at our institution, as only emergent surgery was allowed. This delay created a natural experiment, and it allowed us to perform a prospective study. A previous study examined RCR delays with 6 months of follow-up.^15^ However, studies with longer follow-up results are needed because outcomes after RCR improve beyond 6 months.

Therefore, the purpose of this study was to examine the 2-year outcomes of our prospective group of patients who underwent RCR in a delayed fashion because of the COVID-19 pandemic as compared with those who underwent RCR without a delay. Our hypothesis was that there would not be a notable difference in patient outcomes between the two groups.

## Materials and Methods

### Patient Selection

This was a minimum 2-year follow-up of a prospective clinical study consisting of two groups. (1) Patients planned to undergo arthroscopic RCR for degenerative RCTs between March 16, 2020, and the end of the ban on elective surgery, which lasted 6 weeks at our institution, and (2) patients who underwent arthroscopic RCR for degenerative RCTs beginning 6 weeks after the pause on elective surgery at the University of Utah had been lifted. We omitted individuals who declined preoperative consent and follow-up, those deemed to require urgent or emergent surgical intervention, individuals lacking comprehensive preoperative data, and those undergoing concurrent graft augmentation/interposition or tendon transfer.

### Data Collection

With approval from the institutional review board, informed consent was obtained through telephone. Before surgery, the following outcome measures were gathered: the Simple Shoulder Test (SST), the American Shoulder and Elbow Surgeons (ASES) score, and the visual analog scale (VAS) for pain. Demographic information collected for all patients included age, sex, body mass index, American Society of Anesthesiologists score, smoking status, type of repair construct, concomitant subscapularis repair, biceps treatment, preoperative tear width, preoperative tear retraction, supraspinatus muscle atrophy, infraspinatus muscle atrophy, and the number of anchors used. Subsequently, patients were monitored, and outcome measures were collected preoperatively and at 6 weeks, 3 months, 6 months, and 2 years postoperatively, including the SST, ASES score, and VAS for pain. Magnetic resonance imaging (MRI) was conducted at the 6-month mark to evaluate tendon healing, using protocols similar to previous studies.^16,17^ At 6 months, we previously assessed healing in this cohort through examining MRIs.^15^ Each MRI was evaluated as follows: If there were no fluid-filled gaps between the rotator cuff and the tuberosity on any axial, coronal, or sagittal image, the repair was considered intact. If there was a fluid-filled gap on any image, the repair was considered to be failed.

### Statistical Methods

At 2 years postoperatively, in a randomized clinical trial, the mean ± standard deviation ASES score after primary surgical tendon repair was 93.1 ± 13.9 points.^13^ In a recent study, the minimum clinically important difference (MCID) for the ASES score after arthroscopic RCR was 27.1 points.^18^ Assuming equal variances between groups, a power analysis was conducted, determining that seven patients per group (14 patients total) would be necessary to have 90% chance of finding differences of this magnitude between groups, should one exist. To account for attrition rates, a study with 30 patients (15 per group) was planned.^15^ After 2 years, we obtained 93.3% follow-up with 13 of 15 in the delay group and 15 of 15 in the control group and thus our study was adequately powered for the MCID of our primary outcome. To analyze differences between groups, discrete variables were compared using Chi-square tests and Fisher exact tests as appropriate depending on cell populations, and continuous data were analyzed using Student *t*-tests and Mann-Whitney *U* tests as appropriate depending on data normality as assessed with the Kolmogorov-Smirnov test. Paired data were analyzed using paired Student *t*-tests and related samples Wilcoxon signed rank tests as appropriate based on data normality as above. Interrater and intra-rater reliability were measured using Cohen κ.

## Results

### Patient Demographics

Fifteen patients were included in each group, and we obtained 93.3% follow-up at 2 years with 13 of 15 in the delay group and 15 of 15 in the control group. The delay in surgery was measured from the original surgery date before the 6-week elective surgery ban to the rescheduled date of surgery after the 6-week ban. The time from presentation to surgery was measured from the date the patient first met with the operating surgeon to the date of surgery. The mean ± standard deviation time from presentation to surgery in the control group was 31 ± 17 days while the mean ± standard time from presentation to surgery in the COVID-19 delayed group was 118 ± 80 days. The difference in the average time from presentation to surgery between the two groups was 87 days. There were no significant preoperative differences between groups in any factor, including age (*P* = 0.856), sex (*P* = 0.460), BMI (*P* = 0.751), Charlson Comorbidity Index (*P* = 0.185), American Society of Anesthesiology Score (*P* = 0.964), smoking status (*P* = 1.000), laterality (*P* = 0.882), active forward elevation (*P* = 0.142), active external rotation (*P* = 0.7170), active internal rotation (*P* = 0.345), tear width (*P* = 0.080), tear retraction (*P* = 0.387), or Goutallier grade in the supraspinatus (*P* = 0.125), infraspinatus (*P* = 0.298), or subscapularis (*P* = 0.542, Table 1). Intraoperatively, there were no differences between groups including the number of anchors used (*P* = 0.217), the incidence of concomitant biceps tenodesis (*P* = 1.000), or the incidence of subscapularis repair (*P* = 0.410, Table 2).

**Table 1:** Preoperative Characteristics of Both Groups

Variable	Control (N = 15)	Delay (N = 13)	*P*
Age (yrs)	55 ± 10	55 ± 11	0.856
Time from presentation to surgery (days)	31 ± 17	118 ± 80	0.001
BMI	29 ± 6	29 ± 7	0.751
ASA score	2 ± 1	2 ± 1	0.964
CCI	1.7 ± 1.7	0.9 ± 1.1	0.185
Preoperative AFE	107 ± 56	136 ± 44	0.142
Preoperative ADER	55 ± 23	52 ± 19	0.717
ADIR			
Missing	20% (3)	0% (0)	
Hip	6.7% (1)	0% (0)	
Buttock	13% (2)	8% (1)	
LS Jxn	7% (1)	8% (1)	
L3	40% (6)	31% (4)	
T12	13% (2)	15% (2)	
T7	0% (0)	15% (2)	0.345
Female sex	47% (7)	31% (4)	0.460
Current smokers	0% (0)	0% (0)	1.000
Surgical side dominant	60% (9)	62% (8)	0.882

N/A = not applicable, BMI = body mass index, ASA = American Society of Anesthesiologists, CI = Charlson Comorbidity Index, AFE = active forward elevation, ADER = adducted external rotation, ADIR = adducted internal rotation.

Continuous data are shown as mean ± standard deviation, and discrete data are shown as % (N).

**Table 2:** Intraoperative and MRI Characteristics of Both Groups

Variable	Control (N = 15)	Delay (N = 13)	*P*
Tear width (mm)	16 ± 15	25 ± 16	0.080
Tear retraction (mm)	15 ± 12	20 ± 14	0.387
Number of anchors	2.1 ± 1.4	2.8 ± 1.4	0.217
SS Goutallier			
0	67% (10)	70% (9)	
1	7% (1)	0% (0)	
2	27% (4)	8% (1)	
3	0% (0)	23% (3)	0.125
IS Goutallier			
0	73% (11)	62% (8)	
1	20% (3)	8% (1)	
2	7% (1)	15% (2)	
3	0% (0)	15% (2)	0.298
SSC Goutallier			
0	93% (14)	85% (11)	
1	0% (0)	8% (1)	
2	7%(1)	8% (1)	0.542
Repair construct			
Single row	60% (9)	62% (8)	
Double row	40% (6)	39% (5)	1.000
Biceps tenodesis	87% (13)	85% (13)	1.000
Subscapularis repair	20% (3)	39% (5)	0.410

LS Jxn = lumbosacral junction, SS = supraspinatus, IS = infraspinatus, SSC = subscapularis

Continuous data are shown as mean ± standard deviation, and discrete data are shown as % (N).

### Outcome Scores and Rotator Cuff Tendon Healing

There were significant preoperative versus postoperative differences in ASES scores (*P* < 0.001), VAS scores (*P* < 0.001), and SST scores (*P* < 0.001) but no differences between groups in final outcome scores (*P* = 0.650, 0.586, and 0.525 respectively) (Table 3). In addition, we found no difference in change in VAS or SST preoperative and postoperative in between groups (*P* = 0.467 and 0.363, respectively). We did find a trend toward less improvement in ASES scores in the delay group (*P* = 0.072), but this difference did not reach statistical or clinical significance. A post hoc power analysis concluded that we would need 51 patients per group to find a statistical difference in change in ASES score, should one exist. We had 6-month follow-up MRIs on 83.3% (25/30) of our cohort. Within the delay group, 58.3% (7/12) had healed while within the control group, 84.6% (11/13) had healed (*P* = 0.202). There was an approximately 27% difference in healing rate between the two cohorts. In addition, although there was a trend toward larger tear width in the delay group (*P* = 0.080), this did not reach statistical significance. There were no significant differences between the healed and nonhealed groups in postoperative VAS (*P* = 0.187), SST (*P* = 0.757), or ASES scores (*P* = 0.804) or change in VAS (*P* = 0.951), SST (*P* = 0.260), or ASES (*P* = 0.288).

**Table 3:** Clinical Outcome Scores

Variable	Time Point	Control (N = 15)	Delay (N = 13)	*P*	Number Needed Per Group to Demonstrate a Difference
VAS	Preoperative	5.7 ± 2.1	5.6 ± 2.2	0.935	NA
	2 years postoperative	0.3 ± 0.8	0.9 ± 1.7	0.586	82
	Change	−5.3 ± 2.2	−4.8 ± 2	0.467	292
SST	Preoperative	6 ± 2	5 ± 2	0.211	NA
	2 years postoperative	11 ± 1	12 ± 1	0.525	18
	Change	9 ± 6	11 ± 7	0.363	176
ASES	Preoperative	45 ± 15	48 ± 19	0.624	NA
	2 years postoperative	96 ± 5	91 ± 16	0.65	94
	Change	51 ± 14	42 ± 17	0.072	51

VAS = visual analog scale, NA = not applicable, SST = Simple Shoulder Test score, ASES = American Shoulder and Elbow Surgeons score.

Continuous data are shown as mean ± standard deviation.

## Discussion

This study demonstrated that although preoperative versus postoperative ASES, VAS, and SST scores were significantly improved between preoperatively and postoperatively, there remained no notable differences in final score or score improvement between the delay and control groups at 2 years, despite the approximately 27% higher failure rate in the delay group. Thus, a 3-month nonsurgical treatment period may be reasonable before RCR and may not influence the outcome if RCR is selected later.

Previous literature has suggested that delay in RCR is harmful to outcomes, but the length of the delay necessary to impair outcomes is unclear. In a retrospective analysis of full-thickness RCTs, researchers found no difference in patient-reported outcome measures irrespective of whether the surgical treatment had been performed within 3 weeks, 6 weeks, or 3 months.^10^ Kim et al. conducted a randomized controlled trial of 78 patients with supraspinatus tears and randomized them into two groups with one group receiving immediate RCR and the other receiving delayed RCT after 6 months of nonsurgical treatment. Their study reported higher ASES scores and lower VAS scores in patients who delayed surgical RCR by 6 months at 6 months postoperation.^11^ Interestingly, a systematic review in 2013 found that patients with a delay of greater than 3 months had higher postoperative constant scores.^13^ All these findings are concordant with our own that a brief period of delay less than 3 months may not be harmful.

However, we did find a trend toward less improvement in ASES scores in the delay group. Subsequent studies to examine this effect should have a minimum of 100 patients in both groups combined based on our post hoc power analysis. In addition, although the difference in failure rates at 6 months between groups was not statistically significant (*P* = 0.202), it was numerically large because 15% of RCRs in the control group failed and 42% of RCRs in the delay group failed, i.e. the failure rate was increased over 100% by the delay. Overall, these findings suggest that a delay in treatment may influence failure rates and that more research may be needed. Although there were no differences in outcomes between the healed and nonhealed groups in our study, we may be underpowered to find this difference because the failure rate was low and thus there are not many patients in the retear group. Many insurance companies currently require patients to attempt at least 6 weeks of physical therapy before undergoing surgery. In select patients, this delay in treatment could contribute to increased failure rates. In patients in whom healing is likely to be questionable, particularly large acute tears, more caution should be employed before delaying an RCR.

This study has several limitations. First, the study population was relatively small. Our study was powered to find a difference in ASES of the magnitude of the MCID and is thus adequately powered; however, we may be underpowered for some post hoc analyses. Second, this is a nonrandomized study, and surgical methods were not homogenized in each group. However, all these repairs were performed by two orthopaedic surgeons from the same institution who share similar surgical ideologies, and there were no differences between groups in demographics, tear characteristics, or repair techniques. In addition, at our institution, the delay was performed in a uniform manner in all patients, i.e. no rotator cuff repairs were performed during the 6-week period of delay. However, it is possible that some patients may have injured their shoulder but delayed their presentation during the initial period of the pandemic out of fear of the virus and then presented after elective surgery began again. Theoretically, these patients would then be included in the “control group” despite having a self-induced delay. However, the time between initial presentation and surgery was significantly longer in the delay group than the control group, reducing this risk.

## Conclusion

COVID-19 caused a 3-month delay in the surgical treatment of RCTs. Although this delay showed no statistically significant effect on patient-reported outcome measures, there was an approximately 27% higher failure rate in the delay group. Thus, although a 3-month period of nonsurgical treatment before RCR may be reasonable, given the limitations with respect to the size of our study, these findings are not definitive and warrant future studies with larger sample sizes.
